# Detection of Liver Lesions in Colorectal Cancer Patients Using ^18^F-FDG PET/CT Dual-Time-Point Scan Imaging

**DOI:** 10.3390/cancers15225403

**Published:** 2023-11-14

**Authors:** Luciane G. Boanova, Stephan Altmayer, Guilherme Watte, Ana Amelia Raupp, Martina Zaguini Francisco, Guilherme Strieder De Oliveira, Bruno Hochhegger, Rubens G. F. Andrade

**Affiliations:** 1 Faculty of Medicine, Pontificial Catholic University of Rio Grande do Sul, Av. Ipiranga 6690, Porto Alegre 90619-900, Brazilbhochhegger@ufl.edu (B.H.);; 2 Department of Nuclear Medicine, Hospital Mae de Deus, Av. Jose de Alencar 286, Porto Alegre 90880-481, Brazil; anaaraupp@gmail.com; 3 Graduate Program in Pathology, Federal University of Health Sciences of Porto Alegre, Rua Sarmento Leite 245, Porto Alegre 90050-170, Brazil; g.watte@gmail.com (G.W.); martinazfrancisco@gmail.com (M.Z.F.); 4 School of Medicine, Federal University of Rio Grande do Sul, R. Ramiro Barcelos, 2400—Santa Cecília, Porto Alegre 90035-003, Brazil; guilhermestrdr@gmail.com

**Keywords:** colorectal cancer, colorectal liver metastases, ^18^F-FDG PET/CT, delayed scan, early scan

## Abstract

**Simple Summary:**

Fluorine-18-fluorodeoxyglucose positron emission computed tomography/computed tomography (^18^F-FDG PET/CT) imaging is crucial for staging, restaging, and therapeutic response assessment in colorectal cancer (CRC) patients. However, ^18^F-FDG is not specific for malignant lesions. Dual-time-point imaging has emerged as a promising tool to address this limitation. Our findings indicate that delayed PET/CT images significantly increased both sensitivity (87.7% vs. 100%) and specificity (94% vs. 91%) compared to standard early images. Therefore, our study demonstrated the added value of dual-time-point scans with little extra cost and radiation exposure.

**Abstract:**

Objective: The aim of this study was to evaluate the diagnostic performance of dual-time-point fluorine-18-fluorodeoxyglucose positron emission computed tomography/computed tomography (^18^F-FDG PET/CT) compared to conventional early imaging for detecting colorectal liver metastases (CRLM) in colorectal cancer (CRC) patients. Methods: One hundred twenty-four consecutive CRC patients underwent dual-time-point imaging scans on a retrospective basis. Histopathological confirmation and/or clinical follow-up were accepted as the gold standard. Standard uptake values (SUV), signal-to-noise ratio (SNR), retention index (RI), tumor-to-normal liver ratio (TNR), and lesion sizes were measured for early and delayed PET scans. The diagnostic performance of early and delayed images was calculated on a per-patient basis and compared using McNemar’s test. Results: Among the 124 patients, 57 (46%) had CRLM, 6 (4.8%) had benign lesions, and 61 (49.2%) had no concerning lesions detected. Smaller CRLM lesions (<5 cm^3^) showed significantly higher uptake in the delayed scans relative to early imaging (*p* < 0.001). The SUV and TNR increased significantly in delayed imaging of all metastatic lesions (*p* < 0.001). The retention index of all CRLM was high (40.8%), especially for small lesions (54.8%). A total of 177 lesions in delayed images and 124 in standard early images were identified. In a per-patient analysis, delayed imaging had significantly higher sensitivity (100% vs. 87.7%) and specificity (91.0% vs. 94.0%) compared to early imaging (*p*-value = 0.04). Conclusions: The detection of liver lesions using dual-time-point PET/CT scan improves the sensitivity and specificity for the detection of colorectal liver metastasis.

## 1. Introduction

Colorectal cancer (CRC) accounts for approximately 10% of all annually diagnosed cancers and related deaths worldwide [1]. Nearly 50% of CRC patients will develop colorectal liver metastases (CRLM) during their clinical course [2]. The liver is the most common site of metastasis and represents an important prognostic factor for patients with CRC [3,4]. The current standard of care for CRLM patients involves neoadjuvant chemotherapy and surgical resection combined with adjuvant chemotherapy when feasible, with prognosis largely dictated by the resectability of the metastatic liver disease [3,5,6]. Detecting liver metastasis early has the potential to significantly improve prognosis by more accurately selecting eligible patients for curative hepatic resection and by guiding the optimal choice of treatment strategy [7,8].

Fluorine-18-fluorodeoxyglucose positron emission computed tomography/computed tomography (^18^F-FDG PET/CT) imaging plays an important role in staging, re-staging, and therapeutic response assessment in CRC patients [9,10,11,12]. However, ^18^F-FDG is not specific for malignant lesions. Many benign conditions, inflammatory lesions, and infections can lead to a false-positive finding due to increased ^18^F-FDG uptake [13]. Due to normal intracellular glucose-6-phosphate (G6P) levels in normal tissue, glucose analogues can eventually leave normal cells with time, in contrast to malignant cells that often demonstrate abnormally low levels of G6P [14]. Dual-time-point imaging has emerged as a promising tool to target these differences in physiology between benign and malignant cells, utilizing the dynamic process of ^18^F-FDG uptake and clearance, as tumor cells continue to uptake ^18^F-FDG in delayed imaging, while normal tissue begins clearing the tracer, improving tumor-to-background contrast [14].

Despite advancements in hardware and reconstruction algorithms in recent years, few studies have investigated the clinical value of dual-time-point ^18^F-FDG PET/CT to detect CRLM. Therefore, the aim of this study was to evaluate the diagnostic performance of dual-time-point ^18^F-FDG imaging compared to standard imaging for the detection of CRLM using qualitative and semi-quantitative analysis.

## 2. Materials and Methods

### 2.1. Patient Selection

This retrospective study included 124 consecutive patients who underwent PET/CT scans between June 2020 and January 2021. The inclusion criteria involved patients diagnosed with colorectal cancer who underwent a PET/CT scan for staging or recurrence, based on a primary assessment of the referring physician and available clinical information at the time of the imaging request. All patients had a histological diagnosis of CRC. Patients were excluded from the study if they had (1) previous history of another malignancy, (2) received any therapy in the last 30 days prior to the PET/CT study, or (3) the presence of more than seven lesions.

### 2.2. Reference Standard

The reference standard was based on histopathological analysis whenever available or a combination of clinical follow-up and additional cross-sectional imaging (e.g., CT, magnetic resonance imaging). All patients without a histological diagnosis were followed for up to 12 months and considered true positive if the suspicious lesions significantly regressed or increased in size during a follow-up according to the PERCIST and RECIST criteria [15].

### 2.3. Imaging Protocol

All examinations were performed using a Discovery IQ with 5 rings of detectors (GE Healthcare, Waukesha, WI, USA) PET/CT system with a transaxial field of view of 70 cm and pixel size of 2.73 mm with a matrix size of 256 × 256. The PET imaging system had an axial view of 26 cm per bed position with an inter-slice spacing of 3.75 mm. The patients were asked to fast for at least 6 h prior to the study. Their plasma glucose levels were checked before the injection of ^18^F-FDG and ranged from 73 to 156 mg/dL. All patients received an intravenous ^18^F-FDG injection of 3.7 MBq/Kg body weight (0.10 mCi/Kg) and rested in a waiting room. Initially, low-dose CT imaging was acquired using 120 Kv, 80 mA, and a slice thickness of 5 mm for attenuation correction and anatomical localization with the administration of an oral contrast agent. The standard early whole-body PET/CT was performed from skull to mid-thigh 1 h after injection (62 ± 5 min), and the scan was acquired over approximately 10 min, 2 min per bed position. The delayed emission scanning of the 1-bed position was performed 2 h after injection (122 ± 13 min) over 4 min. All patients were positioned in a supine position with elevated arms. The PET images were reconstructed using the block sequential regularized expectation maximization (BSREM) reconstruction algorithm using a penalty function of 350 (β factor) implemented by GE and known commercially as Q.Clear.

### 2.4. Imaging Data Analysis

The standard early and delayed PET images were evaluated independently by two nuclear medicine physicians with more than 10 years of experience each, and differences were resolved in consensus. A semi-quantitative analysis of the liver lesion uptake using SUV was performed on the GE Advantage Workstation version 4.7. For each patient, a three-dimensional volume of interest (VOI) was carefully drawn on ^18^F-FDG PET/CT-fused images using a fixed threshold of 42% of the SUV_max_ followed by a manual adjustment to exclude the uptake from adjacent tissues to the lesion [16,17]. This procedure was performed for each liver lesion detected on both early and delayed scans to obtain the SUV_max_ and SUV_mean_.

The normal liver tissue parameters (SUV_max-liver_ and SUV_mean-liver_) were obtained through a spherical VOI with a diameter of 3 cm (VOI ≈ 14 cm^3^). The VOI was placed in the center of the right lobe of the liver, avoiding the major blood vessels and liver lesions, and remained the same for both early and late PET imaging.

The signal-to-noise ratio (SNR) in the liver of the standard early and delayed PET/CT scans was measured to compare the image quality of both scans. The SNR was calculated according to the methods previously reported [18,19]. The SNR of the liver in the ^18^F-FDG PET images was defined as the mean of the standardized uptake value (SUV) divided by the standard deviation (SD). The tumor-to-normal liver ratio (TNR) of ^18^F-FDG on both the standard early and delayed scans was calculated for all liver lesions by dividing the maximum lesion SUV of the liver lesion (SUV_max_lesion_) and the mean SUV of normal liver tissue (SUV_mean_liver_) as shown in Equation (1).

(1) $${TNR}_{\left(early,delay\right)}=\frac{{SUV}_{max\_lesion}}{{SUV}_{mean\_liver}}$$

The percentual SUV difference between early and delayed images of all liver lesions was determined by the retention index RI calculated as shown in Equation (2).

(2) $$RI=\frac{{SUV}_{max\_delayed}-{SUV}_{max\_early}}{{SUV}_{max\_early}}\times 100$$

The semi-quantitative analysis of the CRLM lesions was performed for all lesions, lesions with volume smaller than 5 cm^3^ and those larger than 5 cm^3^ for early and delayed PET/CT images.

### 2.5. Statistical Analysis

Data were presented as a mean SD, frequency, percentage, median [IQR], and range. The Shapiro–Wilk test was used to assess the normality of the data distribution. Welch’s *t*-test was used for normal continuous variables, or Wilcoxon signed rank sum test if normality was not met. Pearson’s correlation coefficient was used to assess linear associations between continuous variables. Coefficients were interpreted using the following parameters: 0.00–0.20, very weak; 0.20–0.40, weak; 0.40–0.70, moderate; 0.70–0.90, strong; and 0.90, very strong. The sensitivity, specificity, and accuracy for early and delayed emission scanning were assessed using McNemar’s test in a per-patient analysis. Statistical analyses were performed using R Statistical Software (version 4.2.3, R Core Team 2023, Vienna, Austria).

### 2.6. Ethics Approval

All procedures performed in the studies involving human participants were in accordance with the ethical standards of the institutional and/or national research committee and with the 1964 Helsinki Declaration and its later amendments or comparable ethical standards. The institutional ethics committee of Hospital Mae de Deus approved this study, and all patients gave written informed consent.

## 3. Results

### 3.1. Patient Characteristics

The demographic and clinical characteristics of the included population are presented in Table 1. A total of 124 patients were included with a mean age of 62 ± 12 years and 53 (42.8%) females. The histological classification of the CRC patients of this study was predominantly moderately differentiated (72.6%), followed by well-differentiated (18.5%), and poorly differentiated (8.9%). The median follow-up for all patients was 12 months.

### 3.2. Per-Patient Performance of Early and Delayed Imaging

**Figure 1:**
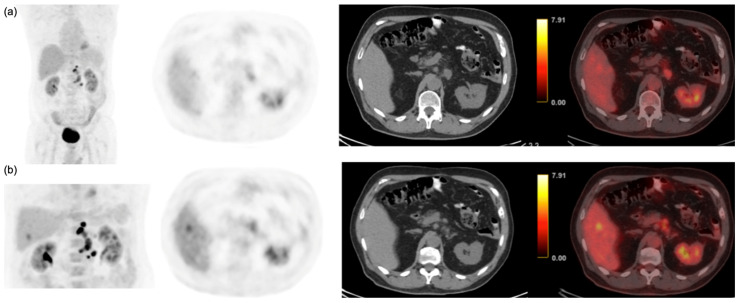
68-year-old patient with colorectal cancer and no liver lesion detected on contrast-enhanced computed tomography. (**a**) Coronal MIP, axial PET, axial CT, and fused CT during the early phase did not demonstrate any hypermetabolic focal liver lesion; (**b**) Coronal MIP, axial PET, axial CT, and fused images during the delayed phase demonstrate a focal hypermetabolic lesion in hepatic segment 5 corresponding to a liver metastasis.

Among the 124 patients, 57 had CRLM, according to the reference standard. Among those, 39 patients (31.2%) had multiple liver lesions, and 24 patients (19.4%) presented only a single lesion. Delayed imaging detected all patients, while early imaging only detected 50 patients. The seven cases only detected in delayed imaging were single liver lesions, as depicted in Figure 1. All the lesions detected in early imaging were also depicted in delayed scans. The per-patient sensitivity of early and delayed PET scans was calculated as 87.7% (50/57) and 100% (57/57), respectively (Table 2).

**Figure 2:**
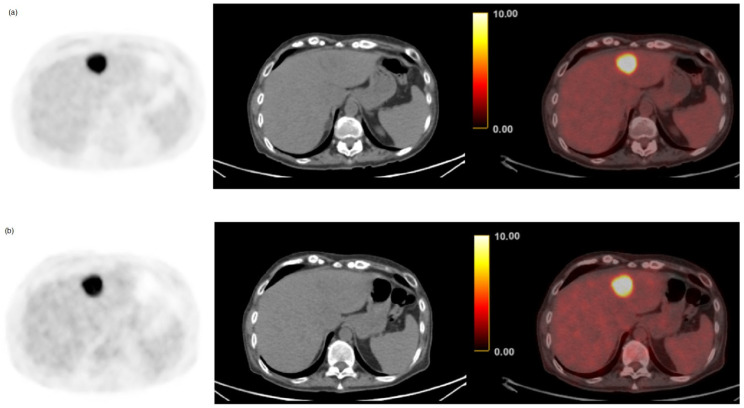
61-year-old female with colorectal cancer and a single hypermetabolic liver lesion in the left lobe on both (**a**) delayed (SUV_max_ 14.3) and (**b**) early phases (SUV_max_ 13.4). Histopathological analysis confirmed the diagnosis of hepatic adenoma.

There were four false-positive cases for metastatic liver disease in the delayed group and six false-positive cases for early imaging, with all cases attributed to a single focal liver lesion. Two false-positive cases were classified based on image follow-up as inflammatory lesions given increased uptake in early imaging with no CT correlation and resolution on follow-up. The remaining four cases were confirmed via biopsy as hepatic adenoma (n = 2), focal nodular hyperplasia (n = 1), and granulomatous disease (n = 1). The per-patient specificity of early and delayed PET was 91.0% (61/67) and 94.0% (63/67), respectively. The McNemar test revealed a significantly higher diagnostic performance of delayed images compared to standard early imaging (*p* = 0.04). A false-positive case of a hepatic adenoma initially interpreted as potential CRLM on both early and delayed imaging is shown in Figure 2.

### 3.3. Semi-Quantitative and Per-Lesion Analysis

**Figure 3:**
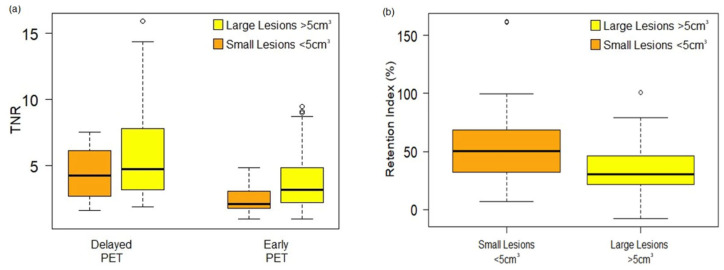
Tumor-to-normal liver ratio and retention index values obtained. (**a**) The TNR in delayed PET scans was significantly higher than in the standard early PET images (*p* < 0.0001), especially in small lesions (*p* < 0.0001); (**b**) The RI of small lesions was significantly higher than large lesions.

The values of normal tissue SUV_max_liver_ and SUV_mean_liver_ in delayed images were significantly lower than in standard early images (Table 3). A total of 177 lesions in delayed images and 124 in standard early images were identified. Altogether, 168 metastatic liver lesions were detected; 160 lesions (92.8%) showed an increase in the SUV_max_ on the delayed scan compared to the early scan and 8 showed no significant change in the SUV_max_. All metastatic lesion SUVs and TNR increased significantly in delayed images when compared to standard early images. The retention index of all CRLM was 40.8% (interquartile range 23.2–55.7%). The TNR and RI exhibited notably higher values in lesions smaller than 5 cm^3^, particularly when compared to larger lesions as demonstrated in Figure 3. The CRLM median lesion volume was 7.1 cm^3^ (interquartile range 3.6–15.4 cm^3^).

The mean value of SNRs in the liver on the delayed PET/CT scan (13.1 ± 2.5) was lower than that on the early whole-body PET scan (13.4 ± 2.8) but not significantly (*p* > 0.05). The SUV_max_, SUV_mean,_ and TNR of small lesions (volume < 5 cm^3^) presented significantly higher uptake in delayed than those on the early scan (*p* < 0.001).

## 4. Discussion

The detection of CRLM using ^18^F-FDG PET/CT dual-time-point scan imaging was enhanced in delayed images when compared to the conventional early imaging protocol. In a per-lesion analysis, delayed images identified 30.1% more lesions than the standard early images, which is likely related to the higher SUV_max_, SUV_mean_, and TNR of metastatic lesions in the delayed imaging. There was also an increase in the sensitivity and specificity of delayed imaging in a per-patient analysis, which is more relevant in the context of CRC due to the significant change in clinical staging in the presence of metastatic disease.

Previous studies using a dual-time-point strategy have demonstrated an improved diagnostic performance compared to early imaging alone. The study conducted by Mao et al. showed a nearly 21% increase in the detection of small CRLM using delayed imaging [20]. Dirisamer et al. also showed a 30% increase in the detection of liver metastasis using a delayed technique in a study including different primary cancers with known liver lesions [21]. Moreover, a recent triple-time-point study by Yen et al. enrolling 310 patients with diverse malignancies for initial staging or recurrence demonstrated a significantly increased sensitivity and accuracy with delayed imaging [22].

The SUV_mean_ and SUV_max_ of normal liver parenchyma in delayed scans were significantly lower compared to early imaging in our study, improving the TNR and RI of hypermetabolic focal liver lesions. Similar results have been described in the literature [20,23,24]. Higashi et al. [25] suggested that a high retention index could predict increased HK-II expression and phosphorylation rate, conditions to accumulate ^18^F-FDG in cancer cells [25]. In our study, the RI of all CRLM was high, especially for small lesions.

Although dual time-point PET scan has been previously investigated by other groups, our study was the first, to our knowledge, to use the Bayesian penalized likelihood (BPL) reconstruction algorithm, which could have contributed to the improved lesion detection. BPL uses a block sequential regularized expectation maximization to improve the algorithm optimization for lesion detection, and its performance is superior to the conventional standard algorithms in small lesion detectability while improving the accuracy of the quantitative parameters [26,27,28]. Parvizi et al. [29] suggested BPL may improve the diagnostic performance of ^18^F-FDG PET/CT in CRLM patients. It uses a penalty function that controls noise suppression, ensuring algorithm convergence of image accuracy and improving the image quality [30]. The algorithm includes point spread function (PSF) modulation that incorporates corrections related to the physical characteristics of radiation interaction in the detection crystals of the PET system. PSF reconstructed images of small lesions can be more susceptible to the Gibbs effect and consequently may overestimate its SUV values, although it improves the small-lesion detectability and spatial resolution [27,31,32]. The partial volume effect (PVE) must also be considered as a factor that can affect the accuracy of the tracer concentration due to the limited spatial resolution of clinical PET systems [33,34].

The SUV values, indicative of tissue ^18^F-FDG uptake, are affected by several factors: patient preparation, physiologic conditions, cross-calibration and clock synchronization between dose calibrator and PET/CT system, data accuracy (weight, height, injection time), image acquisition, and the reconstruction image algorithm. Our study carefully monitored and standardized these variables to ensure the repeatability of the SUV values. Additionally, the SNR_liver_ measurements are influenced by low-count statistics. In our study, the administered dose (calculated per patient based on body weight) and the time from post-injection to early and delayed PET/CT scans were strictly controlled factors; therefore, its influence could be considered negligible. The SNR_liver_ measured in our study was similar in delayed and early images; however, as an index of image quality, SNR is not considered an adequate parameter to be analyzed unattended since image quality is a combination of noise and contrast.

The delayed PET/CT image acquired 2 h after the ^18^F-FDG administration was performed with a 1-bed position localized over the hepatic region to limit the radiation exposure. The incremental radiation dose is minimal using low-dose CT, while the potential benefits of improved sensitivity may have a significant impact on patient management and prognosis [35].

There are several limitations in the present study. Qualitative and semi-quantitative analysis of the ^18^F-FDG PET/CT images is also affected by respiratory motion associated with physiological liver uptake. The SUV_max_ is the most common metric used in clinical practice but is potentially more biased and very sensitive to noise since it is measured from a single voxel within the entire VOI. Although SUV_max_ has limitations, it is more reproducible than SUV_mean_ due to its strong dependence on VOI designing and susceptibility to PVE-induced errors [33,36]. A selection bias due to the inclusion of patients highly suspected of hepatic involvement can be present in the results, and delayed imaging detected all CRLM patients; consequently, the overall sensitivity of delayed PET scans was 100%. Despite the limitations, performing an additional scan for patients suspected to have CRLM supports the potential benefit of improved diagnostic performance.

## 5. Conclusions

The detection of liver lesions in colorectal cancer patients using dual-time-point PET/CT scans improves the detection of metastatic hepatic lesions and specificity compared to the standard early PET/CT scans in patients with colorectal cancer.

## Figures and Tables

**Table 1:** Patient demographics.

Characteristics	n = 124
Age	62.4 ± 12.8
Female	53 (42.8%)
Diabetic Patients	26 (20.1%)
Histological grading of CRC	
Moderately differentiated	90 (72.6%)
Well-differentiated	23 (18.5%)
Poorly differentiated	11 (8.9%)
CRLM	57 (46%)
Benign lesions	6 (4.8%)

CRC, colorectal cancer; CRLM, colorectal cancer liver metastasis.

**Table 2:** Per-patient diagnostic performance of early and delayed imaging for colorectal liver metastasis.

	TP	TN	FP	FN	Sensitivity	Specificity	*p*-Value *	Accuracy
Early	50	61	6	7	87.7%	91.0%	0.04	88.1%
Delayed	57	63	4	0	100.0%	94.0%		96.2%

TP, true positive; TN, true negative; FP, false positive; FN, false negative. * McNemar test for the comparison of diagnostic performance of delayed vs. early imaging.

**Table 3:** Per-lesion characteristics in early and delayed PET imaging.

		Early	Delayed	*p*
Normal liver	SUVmean	2.5 ± 0.4	2.3 ± 0.4	<0.001
	SUV_max_	3.1 ± 0.5	2.8 ± 0.4	<0.001
CRLM	SUVmean	5.3 ± 2.2	5.5 ± 3.0	<0.001
(n = 168)	SUV_max_	8.6 ± 5.4	9.7 ± 7.1	<0.001
	TNR	3.5 ± 2.5	5.4 ± 3.8	<0.001
CRLM > 5 cm^3^	SUVmean	5.9 ± 2.6	7.0 ± 3.7	<0.05
(n = 82)	SUV_max_	10.0 ± 6.4	13.4 ± 8.8	<0.01
	TNR	4.1 ± 2.9	6.1 ± 4.5	<0.001
CRLM < 5 cm^3^	SUVmean	4.4 ± 0.9	5.5 ± 1.5	<0.001
(n = 86)	SUV_max_	6.1 ± 1.7	9.6 ± 3.6	<0.001
	TNR	2.4 ± 0.8	4.3 ± 1.8	<0.001

CRLM, colorectal carcinoma liver metastases; SUV, standard uptake value; TNR, tumor to normal liver ratio; Wilcoxon signed-rank test.

## Data Availability

All data available within the manuscript.

## References

[1] Sung H., Ferlay J., Siegel R.L., Laversane M., Soerjomataram I., Jemal A., Bray F. (2021). Global Cancer Statistics 2020: GLOBOCAN Estimates of Incidence and Mortality Worldwide for Cancers in 185 Countries. CA Cancer J. Clin..

[2] Zhou H., Liu Z., Wang Y., Wen X., Amador E.H., Yuan L., Ran X., Chen W., Wen Y. (2022). Colorectal liver metastasis: Molecular mechanism and interventional therapy. Signal Transduct. Target Ther..

[3] Kow A.W.C. (2019). Hepatic metastasis from colorectal cancer. J. Gastrointest. Oncol..

[4] Zarour L.R., Anand S., Bilingsley K.G., Bisson W.H., Cercek A., Clarke M.F., Coussens L.M., Gast C.E., Geltzeiller C.B., Hansen L. (2017). Colorectal Cancer Liver Metastasis: Evolving Paradigms and Future Directions. Cell. Mol. Gastroenterol. Hepatol..

[5] Dekker E., Tanis P.J., Vleugels J.L., Kasi P.M., Wallace M.B. (2019). Colorectal cancer. Lancet.

[6] Ludwig D.R., Mintz A.J., Sanders V.R., Fowler K.J. (2017). Liver Imaging for Colorectal Cancer Metastases. Curr. Color. Cancer Rep..

[7] Watanabe A., Harimoto N., Araki K., Yoshizumi T., Arima K., Yamashita Y., Baba H., Tetsuya H., Kuwano H., Shirabe K. (2018). A new strategy based on fluorodeoxyglucose-positron emission tomography for managing liver metastasis from colorectal cancer. J. Surg. Oncol..

[8] Hazhirkarzar B., Khoshpuri P., Shaghaghi M., Ghasabeh M.A., Pawlik T.M., Kamel I.R. (2020). Current state of the art imaging approaches for colorectal liver metastasis. Hepatobiliary Surg. Nutr..

[9] Xia Q., Liu J., Wu C., Song S., Tong L., Huang G., Feng Y., Jiang Y., Liu Y., Yin T. (2015). Prognostic significance of ^18^FDG PET/CT in colorectal cancer patients with liver metastases: A meta-analysis. Cancer Imaging.

[10] Agarwal A., Marcus C., Xiao J., Nene P., Kachnic L.A., Subramaniam R.M. (2014). FDG PET/CT in the management of colorectal and anal cancers. AJR Am. J. Roentgenol..

[11] Bijlstra O.D., Boreel M.M.E., van Mossel S., Burgmans M.C., Kapiteijin E.H.W., Oprea-Larger D.E., Rietbergen D.D.D., van Velden F.H.P., Vahrmeijer A.L., Swijnenburg R.-J. (2022). The Value of 18F-FDG-PET-CT Imaging in Treatment Evaluation of Colorectal Liver Metastases: A Systematic Review. Diagnostics.

[12] Viganò L., Lopci E., Costa G., Rodari M., Poretti D., Pedicini V., Solbiati L., Chiti A., Torzilli G. (2017). Positron Emission Tomography-Computed Tomography for Patients with Recurrent Colorectal Liver Metastases: Impact on Restaging and Treatment Planning. Ann. Surg. Oncol..

[13] Metser U., Even-Sapir E. (2007). Increased (18)F-fluorodeoxyglucose uptake in benign, nonphysiologic lesions found on whole-body positron emission tomography/computed tomography (PET/CT): Accumulated data from four years of experience with PET/CT. Semin. Nucl. Med..

[14] Cheng G., Torigian D.A., Zhuang H., Alavi A. (2013). When should we recommend use of dual time-point and delayed time-point imaging techniques in FDG PET?. Eur. J. Nucl. Med. Mol. Imaging.

[15] Wahl R.L., Jacene H., Kasamon Y., Lodge M.A. (2009). From RECIST to PERCIST: Evolving Considerations for PET Response Criteria in Solid Tumors. J. Nucl. Med..

[16] Erdi Y.E., Mawlawi O., Larson S.M., Imbriaco M., Yeung H., Finn R., Humm J.L. (1997). Segmentation of lung lesion volume by adaptive positron emission tomography image thresholding. Cancer.

[17] Boellaard R., Delgado-Bolton R., Oyen W.J.G., Giammarile F., Tatsch K., Eschner W., Verzijlbergen F.J., Barrington S.F., Pike L.C., Weber W.A. (2015). European Association of Nuclear Medicine (EANM). FDG PET/CT: EANM procedure guidelines for tumour imaging: Version 2.0. Eur. J. Nucl. Med. Mol. Imaging.

[18] Maffione A.M., Lopci E., Bluemel C., Giammarile F., Herrmann K., Rubello D. (2015). Diagnostic accuracy and impact on management of (18)F-FDG PET and PET/CT in colorectal liver metastasis: A meta-analysis and systematic review. Eur. J. Nucl. Med. Mol. Imaging.

[19] Amakusa S., Marsuoka K., Kawano M., Hasegawa K., Ouchida M., Date A., Yoshida T., Sasaki M. (2018). Influence of region-of-interest determination on measurement of signal-to-noise ratio in liver on PET images. Ann. Nucl. Med..

[20] Mao W., Zhou J., Qiu L., Yin H., Tan H., Shi H. (2020). The added value of dual-time-point 18F-FDG PET/CT imaging in the diagnosis of colorectal cancer liver metastases. Abdom. Radiol..

[21] Dirisamer A., Halpern B.S., Schima W., Heinisch M., Wolf F., Beheshti M., Dirisamer F., Weber M., Langsteger W. (2008). Dual-time-point FDG-PET/CT for the detection of hepatic metastases. Mol. Imaging Biol..

[22] Yen Y.-A., Huang W.-S., Chiu C.-H., Tyan Y.-C., Wang J.-Y., Wu L.-C., Feng I.J., Lee C.H. (2020). Does Routine Triple-Time-Point FDG PET/CT Imaging Improve the Detection of Liver Metastases?. Diagnostics.

[23] Kubota K., Itoh M., Ono S., Tashiro M., Yamaguchi K., Akaizawa T., Yamada K., Fukuda H. (2001). Advantage of delayed whole-body FDG-PET imaging for tumour detection. Eur. J. Nucl. Med..

[24] Lee J.W., Kim S.K., Lee S.M., Moon S.H., Kim T.S. (2011). Detection of hepatic metastases using dual-time-point FDG PET/CT scans in patients with colorectal cancer. Mol. Imaging Biol..

[25] Higashi T., Saga T., Nakamoto Y., Ishimori T., Mamede M.H., Wada M., Doi R., Hosotani R., Imamura M., Konishi J. (2002). Relationship Between Retention Index in Dual-Phase 18 F-FDG PET, and Hexokinase-II and Glucose Transporter-1 Expression in Pancreatic Cancer. J. Nucl. Med..

[26] Te Riet J., Rijnsdorp S., Roef M.J., Arends A.J. (2019). Evaluation of a Bayesian penalized likelihood reconstruction algorithm for low-count clinical 18F-FDG PET/CT. EJNMMI Phys..

[27] Rogasch J.M.M., Hofheinz F., van Heek L., Voltin C.-A., Boellaard R., Kobe C. (2022). Influences on PET quantification and interpretation. Diagnostics.

[28] Liu Y., Gao M.-J., Zhou J., Du F., Chen L., Huang Z.-K., Hu J.-B., Lou C. (2021). Changes of [18F]FDG-PET/CT quantitative parameters in tumor lesions by the Bayesian penalized-likelihood PET reconstruction algorithm and its influencing factors. BMC Med. Imaging.

[29] Parvizi N., Franklin J.M., McGowan D.R., Teoh E.J., Bradley K.M., Gleeson F.V. (2015). Does a novel penalized likelihood reconstruction of 18F-FDG PET-CT improve signal-to-background in colorectal liver metastases?. Eur. J. Radiol..

[30] Van der Vos C.S., Koopman D., Rijnsdorp S., Arends A.J., Boellaard R., van Dalen J.A., Lubberink M., Willemsen A.T.M. (2017). Quantification, improvement, and harmonization of small lesion detection with state-of-the-art PET. Eur. J. Nucl. Med. Mol. Imaging.

[31] Devriese J., Beels L., Maes A., Van de Wiele C., Pottel H. (2018). Impact of PET reconstruction protocols on quantification of lesions that fulfil the PERCIST lesion inclusion criteria. EJNMMI Phys..

[32] Munk O.L., Tolbod L.P., Hansen S.B., Bogsrud T.V. (2017). Point-spread function reconstructed PET images of sub- centimeter lesions are not quantitative. EJNMMI Phys..

[33] Zaidi H., Alavi A., Naqa I.E. (2018). Novel Quantitative PET Techniques for Clinical Decision Support in Oncology. Semin. Nucl. Med..

[34] Meikle S.R., Sossi V., Roncali E., Cherry S.R., Banati R., Mankoff D., Jones T., James M., Stutcliffe J., Ouyang J. (2021). Quantitative PET in the 2020s: A roadmap. Phys. Med. Biol..

[35] Tian A., Lin R., Yu J., Zhang F., Zheng Q., Yuan X., Sun Z., Zhong Z. (2022). The differential diagnostic value of dual-phase 18F-DCFPyL PET/CT in prostate carcinoma. Prostate Cancer Prostatic Dis..

[36] Zaidi H., Karakatsanis N. (2018). Towards enhanced PET quantification in clinical oncology. Br. J. Radiol..

